# Optical spectral diagnostics of the oxygenation level in periodontal tissues and photodynamic therapy using methylene blue in children with cerebral palsy

**DOI:** 10.3389/fpubh.2023.961066

**Published:** 2023-01-30

**Authors:** Natalia S. Morozova, Iuliia A. Kozlitina, Vladimir I. Makarov, Victor B. Loschenov, Vasiliy M. Grinin, Sergey Yu. Ivanov, Maria S. Kashtanova

**Affiliations:** ^1^Department of Pediatric Dentistry and Orthodontics, I.M. Sechenov First Moscow State Medical University (Sechenov University), Moscow, Russia; ^2^Laser Biospectroscopy Laboratory, Prokhorov General Physics Institute of the Russian Academy of Sciences, Moscow, Russia; ^3^Department of Laser Micro-, Nano- and Biotechnologies, Institute for Physics and Engineering in Biomedicine, National Research Nuclear University MEPhI (Moscow Engineering Physics Institute), Moscow, Russia; ^4^Laboratory of Laser Biospectroscopy, Prokhorov General Physics Institute of the Russian Academy of Sciences, Moscow, Russia; ^5^Department of Maxillofacial Surgery, I.M. Sechenov First Moscow State Medical University (Sechenov University), Moscow, Russia; ^6^Department of Maxillofacial Surgery, The Peoples' Friendship University of Russia, Moscow, Russia

**Keywords:** optical spectral diagnostics, photodynamic therapy, phototheranostic, periodontal disease, gingivitis, children with cerebral palsy, methylene blue

## Abstract

Dental diseases occur in children with cerebral palsy three times higher than in healthy children. Low values of the unstimulated salivation rate (<0.3 ml per minute), pH and buffer capacity, changes in enzyme activity and sialic acid concentration, as well as increased saliva osmolarity and total protein concentration, which indicates impaired hydration, are the factors in the development of a gingiva disease in case of cerebral palsy. This leads to increased bacterial agglutination and the formation of acquired pellicle and biofilm, leading to the formation of dental plaque. There is a tendency toward an increase in the concentration of hemoglobin and a decrease in the degree of hemoglobin oxygenation, as well as an increase in the generation of reactive oxygen and nitrogen species. Photodynamic therapy (PDT) with the use of photosensitizer methylene blue improves blood circulation and the degree of oxygenation in periodontal tissues, as well as eliminates a bacterial biofilm. Analysis of back diffuse reflection spectra makes it possible to conduct non-invasive monitoring determine tissue areas with a low level of hemoglobin oxygenation for precision photodynamic exposure.

**Aim:**

To improve the effectiveness of phototheranostics methods using, namely PDT with simultaneous optical-spectral control, for the treatment of gingivitis in children with complex dental and somatic status (cerebral palsy).

**Methods:**

The study involved 15 children (6-18 y.o.) with various forms of cerebral palsy, in particular, spastic diplegia and atonic-astatic form and with gingivitis. The degree of hemoglobin oxygenation was measured in tissues before PDT and on the 12th day. PDT was performed using laser radiation (λ = 660 nm) with a power density of 150 mW/cm^2^ with a five-minute application of 0.01% MB. The total light dose was 45 ± 15 J/cm^2^. For statistical evaluation of the results, a paired Student's t-test was used.

**Results:**

The paper presents the results of phototheranostics using methylene blue in children with cerebral palsy. An increase in the level of hemoglobin oxygenation from 50 to 67% (*p* < 0.001) and a decrease in blood volume in the microcirculatory bed of periodontal tissues were shown.

**Conclusion:**

Photodynamic therapy methods with application of methylene blue make it possible to assess the state of the gingival mucosa tissue diseases objectively in real time, and to provide effective targeted therapy for gingivitis in children with cerebral palsy. There is a prospect that they can become widely used clinical methods.

## Introduction

Cerebral palsy (CP) is a common (2.0–2.5 cases per 1,000) chronic movement disorder, often accompanied by impaired coordination, cognitive functions, communication and seizure disorders (1–3). These disorders, as well as medications used by children with CP (4), have a direct impact on the development of various diseases, but one of the most common pathologies in case of cerebral palsy, affecting the child's quality of life, are dental or oral diseases (5, 6). All research groups that studied the prevalence of diseases of periodontal and dental hard tissues in children found that children with CP had the prevalence of dental diseases in three times higher than in the control group (7). The more severe the degree of neurological stroke was and, as a result, cognitive and motor deficits, the higher the risk of developing dental diseases (8–11). Inability to maintain oral hygiene, difficulty in chewing and swallowing, excessive drooling (sialorrhea), recurrent regurgitation and vomiting, insufficient calcium intake, vitamin D deficiency, use of antiepileptic medications are only a part of the factors leading to the onset and development of dental pathologies in children with CP (12–16). Along with caries, gingivitis is observed in the vast majority of children with CP (17–19). Low indices of the unstimulated salivation rate, pH and buffer capacity, changes in enzyme activity and sialic acid concentration, as well as increased saliva osmolarity and total protein concentration, which indicates impaired hydration, are the factors in the development of gingiva diseases in case of cerebral palsy (20, 21). This leads to increased bacterial agglutination and the formation of acquired pellicle and biofilm, leading to the formation of dental plaque (22).

The inflammatory response of the gingivas after the onset of the plaque formation is characterized by a change in vascular morphology that precedes clinical changes. As the inflammation of the gingivas increases, the expansion and proliferation of the gingiva vessels is observed, as well as their course changes and the number of functioning units increases. However, as shown in the study (23) of the degree of oxygenation of the inflamed periodontal tissues, the increase in blood supply is insufficient to meet the oxygen demand of the inflamed gingivas, in which the Hb index (tissue blood filling) was significantly higher than in clinically healthy gingivas. A lower level of tissue SO_2_ was observed in the inflamed area. Hypoxia and inflammation are usually closely related (24). Hypoxia regulates vascular tone and is a powerful stimulus for angiogenesis (25), which ensures the supply of oxygen and nutrients to tissues, and restores homeostasis during wound healing (26). Endothelial cells are located at the boundary between blood and tissues and their function directly depends on changes in oxygen tension (27). Endothelial cells respond to hypoxia through the expression of regulatory genes mediated by various oxygen-dependent signaling cascades (28, 29). Hypoxia-inducible factor (HIF) is a key regulatory protein for hypoxia-mediated events (30). It regulates the expression of many genes involved in adaptation to oxygen deficiency, including cell proliferation, apoptosis, metabolism, immune responses, genomic instability and vascularization, the expression of VEGF, nitric oxide synthase (NOS), and the release of cytokines that regulate angiogenesis (31).

The bacterium *F. nucleatum* plays a special role in the progression of periodontitis and gingivitis (32). It stimulates pro-inflammatory changes in endothelial cells (33, 34) and induces hypoxia by itself, reducing the oxygen content in the environment (35, 36). As a result, endothelial cells demonstrate decreased expression of CD31 and increased CD34 (37), which leads to endothelial dysfunction, increased inflammation, and increased activation of T-cells (38). Another inflammatory factor during hypoxia is an increase in the immunoreactivity of NF-kB, HIF-1 and VEGF, as well as an increase in the expression of IL-1β and MMP-1 in accordance with the progression of diseases (39), which induces vascular permeability and stimuli of invasion of immune cells, such as as monocytes/macrophages and neutrophils, which contribute to the destruction of periodontal tissue.

In modern clinical practice, the diagnosis of periodontal disease is reduced to asking about complaints, writing a case history, instrumental examination, radiography. The evaluation is carried out taking into account the condition of soft tissues, the integrity of the epithelial attachment, the presence and depth of periodontal pockets, the degree of tooth mobility. The Green Vermillion Hygiene Index (OHI-S), the Muehlemann Bleeding Index (SBI), the Schiller-Pisarev test, the papillary-marginal-alveolar index (PMA), and others are commonly used. The general drawback of these methods lies in the fact that the degree of the disease is determined visually, therefore, it is difficult to get rid of the problem of conventionality of digital values determined by a doctor. These methods do not give an opportunity to evaluate the dynamic processes occurring in the tissues and do not allow us to determine the degree of hemoglobin oxygenation in the microvasculature and the level of blood flow in the tissue. Since today microbial factors (recognized by WHO) are responsible for inflammatory-destructive periodontal lesions, the choice of etiotropic therapy of inflammatory periodontal diseases is carried out on the basis of bacteriological examination of the contents of gingival pockets, giving an opportunity to eliminate or weaken its influence by the subsequent use of an etiotropic complex (40). This complex usually provides the direct removal of a biofilm and hard dental plaque; the use of agents that suppress both the maturation of the biofilm and the degree of its pathogenic effect on tissues—antiseptics, and, if necessary, antibiotics. However, more and more antibiotic-resistant bacteria species are emerging, which complicate the possibility of drug treatment, and standard therapies are no longer effective. The use of photodynamic therapy (PDT) with photosensitizers (PS) (41, 42) may help to overcome these difficulties in the treatment of periodontal diseases.

PDT is a method used as a therapy for oral diseases of various origins, such as oncology, precancerous diseases, caries, periodontitis, and gingivitis (43). The advantages of this method are multifactorial effects, antibacterial, anti-inflammatory and wound healing (44). Methylene blue (45), phthalocyanines (46), chlorins (47), porphyrins, etc. are usually used for antimicrobial PDT.

The effect of MB on the regulation of inflammatory processes has been studied (48). It has been shown that MB attenuates the activation of inflammasomes that induce maturation of IL-1β ℵ IL-18, and also a protein such as caspase-1, which mediates cell death known as apoptosis, is part of the inflammasome. In addition, MB inhibits the initial signal of activation of the inflammatory process, phagocytosis, and expression of genes of inflammatory components by inhibiting NF-kB signaling.

The use of PDT with MB in severe chronic periodontitis showed that clinical variables showed significant improvement in IL-1α, IL-1β, IL-8, IL-1ra, IFN-γ, IL-10 and VEGF (49).

**Figure 1:**
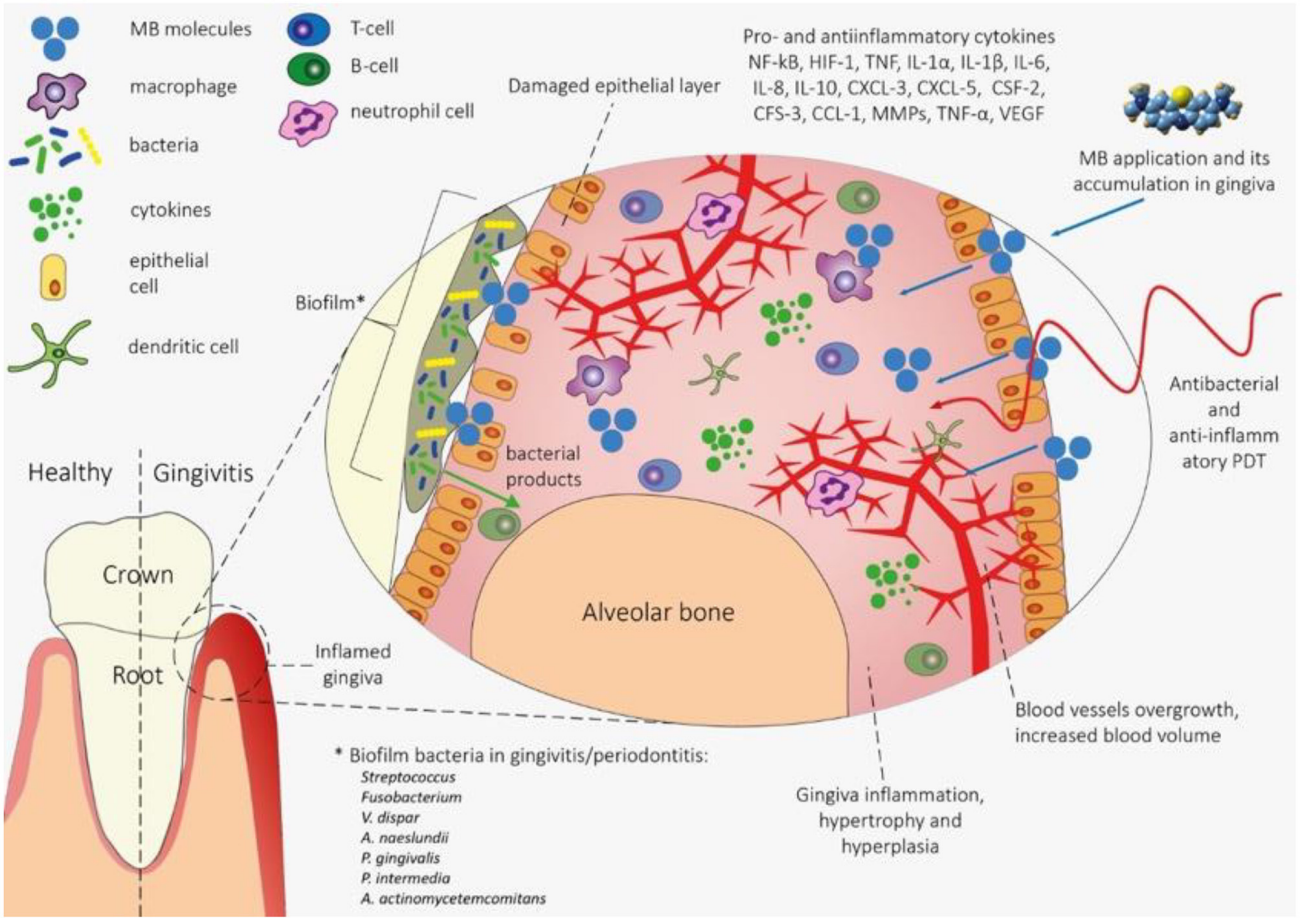
The mechanism of PDT effects on gingiva tissue in gingivitis. The waste products of biofilm bacteria lead to functional disruption and death of epithelial cells, which contributes to the onset of the inflammatory process triggered by immunocompetent cells (macrophages, neutrophils and T cells). There is increased blood flow, decreased hemoglobin oxygenation and gingiva hyperplasia. When MS is applied to gingival tissue, bacteria and gingival tissue cells are sensitized with a photosensitizer (primarily phagocytes). With the help of photodynamic action, bacteria are eliminated in the biofilm and pro-inflammatory macrophages are deactivated, which leads to a decrease in inflammation and normalization of blood flow and the degree of hemoglobin oxygenation. Methylene blue (MB) is of greatest interest as a PS for anti-inflammatory PDT in order to increase the degree of hemoglobin oxygenation in the microvasculature of periodontal tissues. Initially, methylene blue was the first stain used in medicine as an antiseptic and the first photosensitizer tested and approved for use for antimicrobial photodynamic therapy (50).

The explanation of the mechanism of anti-inflammatory photodynamic action in gingivitis is a non-trivial task (Figure 1). Biofilm products secreted by bacteria, such as endotoxins, trypsin-like enzymes, acid and alkaline phosphatases, etc. (51) induce functional disorders and death of gingival epithelial cells. As a result, a cascade inflammatory response is induced, triggered by resident macrophages, neutrophils, and T-cells (52). Inflammation in periodontal pockets is characterized by low oxygen levels and an acidic medium, and mechanical stress caused by chewing, rubbing, or orthodontic treatment can provoke deformation of the blood vessels, contributing to ischemia and hypoxia (53).

This leads to an increase in the immunoreactivity of NF-kB, HIF-1, VEGF, IL-1β and MMP-1 and promotes the invasion of immune cells such as monocytes and/or macrophages and neutrophils. Immune cells, in turn, express IL-8, TNF, IL-6, IL-1β, and IL-10, and epithelial cells show elevated regulation of IL-1α, CXCL-3, CXCL-5, CSF-2, CFS- 3, CCL-1 (54).

The use of PDT with MB can deactivate inflammation and completely eliminate bacteria in the biofilm (55), which will contribute to the functional normalization of the gingiva tissue and the restoration of oxygenation in the microvasculature (56, 57).

Thus, to control PDT of gingivitis, it is necessary to monitor the degree of hemoglobin oxygenation and the level of hemoglobin oxygenation in the microvasculature of periodontal tissues. Optical methods are best suited for these purposes, since they are non-invasive, fast and relatively cheap. In modern clinical dentistry, hemodynamic parameters can be determined using laser Doppler flowmetry (LDF) (58–60) laser speckle contrast imaging (LSCI) (61), Doppler OCT and using backsward diffuse reflection spectroscopy (BDR) (23, 62). Compared to single-point laser Doppler flowmetry (LDF), the LSCI is capable of showing multiple examined regions rather than one. Another unique feature of LSCI is its fast imaging, which reduces movement artifacts and shortens the time of each measurement session. The drawback of LDF is its high sensitivity to the position of the sensor relative to the measured area, which complicates the processing of the results. The measurement results also depend not only on the blood flow in the measured area, but also on the scattering properties of the surrounding tissues. Another limitation of the LDF method is the lack of absolute zero measurement, even if the flow of red blood cells is reduced to zero experimentally or surgically. This is explained by the Brownian motion of macromolecules in the interstitial compartment (63). As a result, changes in gingival blood flow cannot be compared between patients, and values of blood flow at different sites cannot be compared even in the same patient. The main shortcoming of the LSCI methods is the low probing depth (up to 300 microns) due to a loss of light coherence during dispersion in biological tissue. With the help of OCT one can only determine the structure of blood vessels without dynamic parameters. Doppler OCT can only measure blood flow velocity in large vessels where pulsating blood flow is observed. Thus, diffuse backscatter spectroscopy, the probing depth of which reaches several millimeters, and also allows one to determine the degree of hemoglobin oxygenation and the level of blood circulation in the microvasculature of tissues, is the most preferred method for assessing the effectiveness of photodynamic effects in case of gingivitis. BDR spectroscopy is also well combined with fluorescence spectroscopy, which makes it possible to determine the PS concentration in tissues before and after PDT (64).

## Purpose

To improve the effectiveness of the gingivitis treatment in children with complicated dental and somatic status (cerebral palsy), based on the use of photodynamic therapy with the simultaneous use of optical diagnostic methods.

## Materials and methods

### The patients

We analyzed the results of therapeutic treatment for catarrhal gingivitis in 15 patients with various forms of cerebral palsy, in particular, spastic diplegia and atonic-astatic form. The patients were in the 6-18 age range with a male predominance (10:5).

### Optical spectral assessment of tissue oxygenation

**Figure 2:**
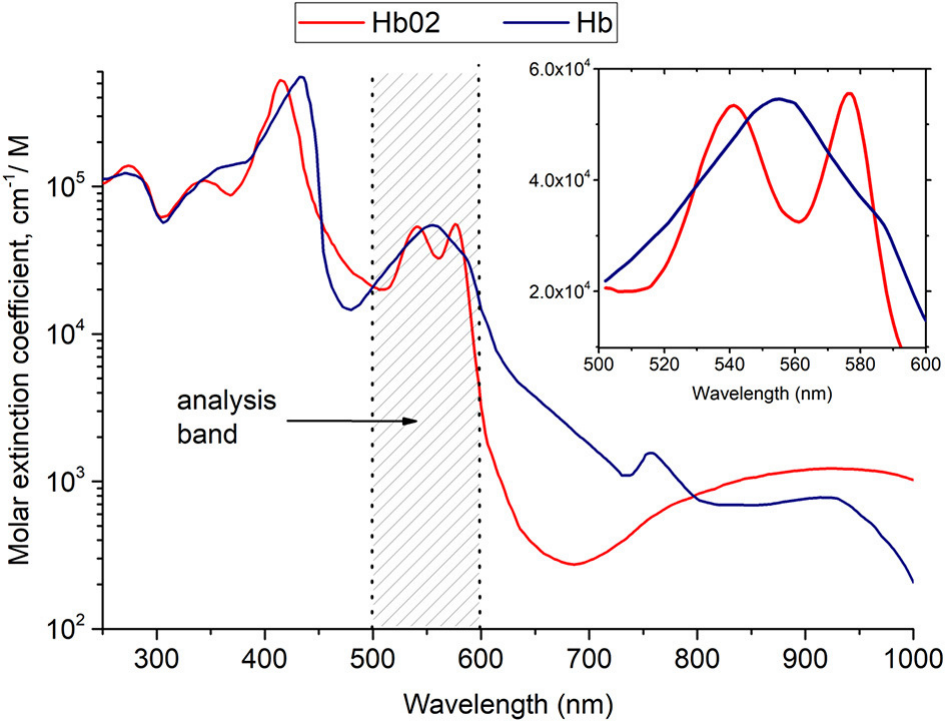
Results of hemoglobin oxygenation measurement in the microcirculatory supply of periodontal tissues.

To quantify the degree of oxygenation and the relative amount of hemoglobin in the tissue, the spectroscopic method was used, which was described in detail in the works of researchers (65, 66) (see Figure 2). The principle of this method consists in analyzing the logarithm of the diffuse reflectance spectrum in the spectral range of 500–600 nm, where the shapes of the absorption spectra of hemoglobin in oxygenated and reduced forms have the greatest differences.

To measure the absorption spectra of periodontal tissues, we used an LESA-01-BioSpec fiber-optic spectrometer with a spectral resolution of 8 nm and an entrance slit of 200 μm. A halogen lamp with a fiber optic output was used as a source of broadband radiation for recording absorption spectra. A fiber-optic probe with a central illumination fiber was used to provide delivery and reception of radiation, which supplied either laser radiation exciting fluorescence or broadband radiation to the tissue, and six peripheral fibers collecting radiation. The measurement of spectra is carried out in gentle contact (without pressure) with the biological tissue to exclude the Fresnel reflection of light coming from the surface of the biological tissue from getting into the receiving fibers and to avoid the displacement of blood from the examined area. For each patient, an area with clinically pronounced gingivitis was selected, as well as an area of conditionally healthy tissue. From 5 to 10 measurements of back diffuse reflection spectra were measured in each area, then the results were averaged for each area of each patient. For statistical evaluation of the results, a paired Student's *t*-test was used.

The obtained spectra were processed using the UnoMomento software (BioSpec LLC).

### Photodynamic therapy using MB

The MB solution was 0.01% MB (absorption maximum λ = 662 nm). PS was applied for 5 min. An LFT-02-Biospec semiconductor laser with a wavelength λmax = 660 nm was used as a radiation source for photodynamic exposure. Laser radiation was delivered to the PDT area using a fiber-optic bundle with a microlens, which provided distinct boundaries of the exposure spot. The power density of the exposure for each selected area of tissue was from 100 to 200 mW/cm^2^, the exposure time was 5 minutes, the light dose was 45 ± 15 J/cm^2^.

## Results

**Figure 3:**
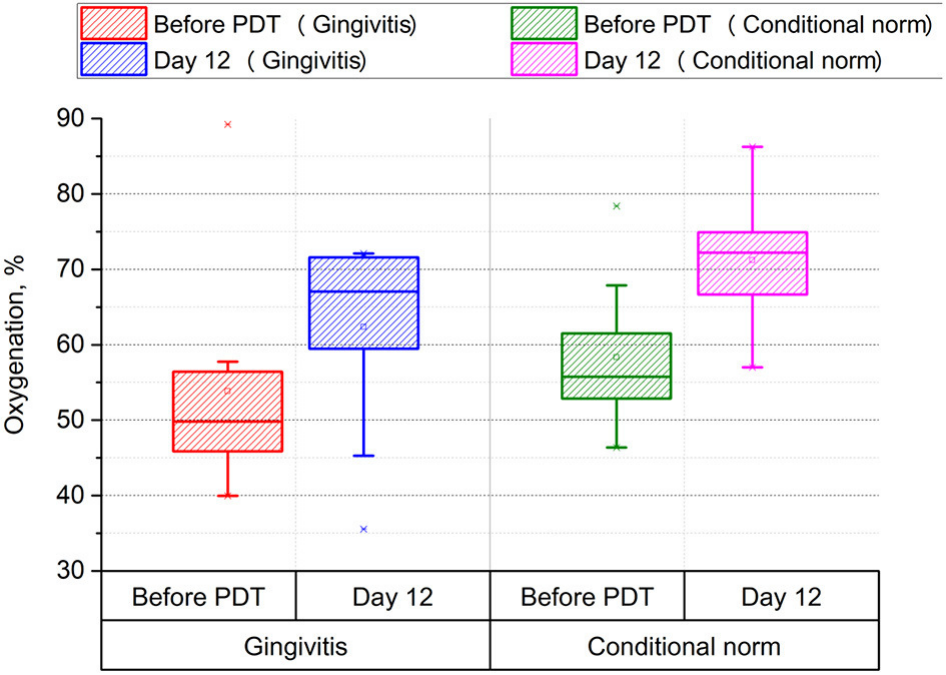
Assessment of the level of gingival tissue oxygenation before and after photodynamic therapy (*p* < 0.001, power > 0.99).

These results testify to a positive anti-inflammatory effect of MB and high-quality treatment using PDT and MB. Figure 2 shows the results of hemoglobin oxygenation in the microvasculature of tissues (see Figure 3).

The use of PDT in treatment leads to a significant increase in oxygenation both in the area affected with gingivitis (from 50 to 67%, *p* < 0.001) and in the conditionally healthy area of periodontal tissue (from 57 to 72%, *p* < 0.001), which indicates a positive effect of PDT on energy and metabolic processes in periodontal tissues.

## Discussion

The difficulty of performing most dental procedures in children with CP is beyond doubt. Almost all dental procedures in these patients involve sedation and anesthesia. Children with CP may be difficult to handle and uncooperative during dental examination and treatment. Therefore, in this case, non-invasive methods of exposure, such as phototheranostics, are of particular relevance. Unfortunately, at the moment in scientific practice there are practically no studies on the use of PDT and optical-spectral diagnostics in patients with cerebral palsy. First of all, this is due to the difficulties of obtaining all permits for conducting these studies.

Although in the world practice extensive research is being carried out on the possibility of using photodynamic therapy for diseases of the oral cavity (67), our scientific group for the first time applied phototheranostics methods in children with cerebral palsy.

The choice of this particular treatment method is due to the multifactorial nature of the photodynamic effect: antibacterial (68), anti-inflammatory (69) and wound healing (70), and the fact that phototherasnostics is a non-invasive and conservative method with few side effects (71). Another advantage of phototheranostics is its minimally invasive action, which can be especially sought after by children, youth and adults who are afraid of dental procedures. Considering that many children and patients with special needs find it difficult to cooperate during long-term clinical dental treatment, the use of PDT in the field of pediatric dentistry and in patients with special needs is very promising. Because it is a painless treatment with no unpleasant taste, it allows the clinician to have regular consultations (72).

Phototheranostics methods for the treatment and prevention of periodontal isease can be non-invasive, and therefore well suited for use in such patients. The widespread introduction of phototheranostics methods is especially important for patients who are unable to independently monitor the health of the oral cavity and tolerate standard treatment methods due to the limited choice of specific dental tools and diagnostic and treatment methods that ensure rapidity, non-invasiveness and effectivenessThe results of our study showed a significant increase in the oxygenation of biological tissues (from 50 to 67%) as a result of photodynamic exposure with a five-minute application of 0.01% MB solution. This results provide prerequisites for the development of new clinical methods that allow non-invasive and painless, real-time assessment of the periodontal diseases state, as well as effective therapy for gingivitis in children with cerebral palsy.

Our future research will focus on obtaining data on biochemical parameters and indicators of inflammation obtained as a result of the work that will allow a deeper understanding of the mechanisms of photodynamic effects on tissues, which can allow the development of algorithms for the clinical application of photodynamic therapy using the photosensitizer methylene blue not only for periodontal diseases, but also for other inflammatory diseases.

## Conclusion

At present, the clinical experience that has been accumulated in the world is not sufficient to formulate the optimal tactics for treating children with cerebral palsy. It is necessary to select an individual approach to such patients, and, as a rule, making emphasis on more comfortable treatment.

Children with cerebral palsy are at increased risk of developing dental problems compared to healthy children, which can lead to their increased incidence and prevalence and significantly affect children's well-being and quality of life.

The conducted study showed that the use of photodynamic therapy with methylene blue gives reliable results in the treatment of children with cerebral palsy. There was a trend toward an increase in tissue saturation on the 12th day after photodynamic exposure (from 50 to 67%).

The indisputable advantage of the methylene blue is that this drug has already been approved for use in pediatric dentistry, which means that the possibility of a wide practical implementation of the results seems very likely. Methods of phototheranostics using methylene blue make it possible to objectively assess in real time the state of the gingival mucosa tissues diseases, and to provide effective targeted therapy for gingivitis in children with cerebral palsy and can become promising clinical methods.

## Data availability statement

The raw data supporting the conclusions of this article will be made available by the authors, without undue reservation.

## Ethics statement

The studies involving human participants were reviewed and approved by Local Ethics Committee FSAEI HE I. M. Sechenov First Moscow State University of the Ministry of Health of the Russian Federation (Sechenov University). The patients' guardian provided their written informed consent to participate in this study.

## Author contributions

All authors listed have made a substantial, direct, and intellectual contribution to the work and approved it for publication.
